# Case report: Escitalopram-associated lower limb edema

**DOI:** 10.3389/fpsyt.2024.1394813

**Published:** 2024-04-26

**Authors:** Mohamed Hassan Ahmed, Mena Al-Kubaisi, Safa Abdulmajeed Al-Rawi, Omar Hosam Salama, Hebah Mahmoud Abutayyem

**Affiliations:** ^1^ Consultant Psychiatrist, Sheikh Khalifa Medical City, Ajman, United Arab Emirates; ^2^ Bachelor of Medicine and Bachelor of Surgery (MBBS), Ajman University, Ajman, United Arab Emirates; ^3^ Pharmacy Department, Sheikh Khalifa Medical City, Ajman, United Arab Emirates

**Keywords:** edema, drug-associated, side effects, SSRIs, escitalopram

## Abstract

Escitalopram is widely prescribed for the treatment of major depressive disorder and generalized anxiety disorder with a well-documented side effects profile. Peripheral edema, however, is a rarely reported adverse reaction that warrants further work up. This paper summarizes the case of a 58-year female patient who developed transient bilateral peripheral edema following the administration of low dose escitalopram. This case underscores the necessity for clinicians to be familiar with even rare potential side effects of commonly prescribed medications. It also suggests a need for patient education regarding the importance of reporting new symptoms promptly.

## 1 Introduction

Escitalopram, a selective serotonin reuptake inhibitor (SSRI), is commonly utilized in the treatment of major depressive disorder and generalized anxiety disorder due to its efficacy and relatively favorable side effect profile (1). Despite its widespread use, certain adverse drug reactions (ADRs) remain less characterized in the medical literature. Peripheral edema is infrequently associated with SSRI (1). Peripheral edema typically presents as a swelling in the lower extremities. It has been associated with a myriad of etiologies, including cardiac, renal, hepatic, or venous insufficiency, as well as several pharmacological agents (2). The incidence of drug-associated peripheral edema is often underreported and may lead to non-compliance or unnecessary medical investigations if not promptly recognized (2). We report a case of a 58-year-old-year-old female patient who developed transient bilateral peripheral edema following the administration of escitalopram. The case presents a unique instance of an adverse drug reaction (ADR) to a widely used medication, distinguished by the absence of other typical causes of edema. This is confirmed through extensive diagnostic tests. Importantly, the connection between the medication and edema is underscored by the precise timing of the reaction and the swift resolution after stopping the medication, which substantiates a direct relationship between the two. Through this report, we aim to highlight the clinical approach to diagnosing and managing such an atypical presentation and discussing the broader implications on management with psychotropic medications (3).

## 2 Case presentation

A 58-year-old female, previously healthy with unremarkable medical and family history, presented with disturbed sleep. She gradually developed insomnia and appetite loss over one month, prior to her visit, and she also suffered from low mood. A full medical assessment was done in a private hospital which indicated no underline medical explanation for her symptoms. A plan was made, and she was provided with psychoeducation and a therapeutic regimen of escitalopram, initiated at a dose of 5 mg/day, for 30 days, to manage her depressive episode. Later, she presented to our outpatient department for follow up with an acute onset of bilateral lower limb swelling. This event occurred after 6 days from the commencement of the therapeutic regimen. Notably, the patient had not reported any recent alterations in her medication regimen or any significant medical history that could contribute to the current symptomatology. Upon examination, the patient exhibited bilateral peripheral edema, characterized by swelling extending from the dorsum of the feet to the mid-calves. The edema was pitting, without accompanying erythema, ulceration, or discoloration of the overlying skin, which may have suggested an inflammatory or infectious etiology. Her cardiovascular assessment did not reveal any signs suggestive of congestive heart failure, and her abdominal examination was unremarkable, discounting hepatic or renal pathology as a primary cause.

## 3 Diagnostic assessment

Laboratory investigations were promptly conducted, which included a complete blood count, renal function panel, which included electrolytes such as calcium, sodium, potassium and chloride, liver function panel, thyroid function tests, and a comprehensive urinalysis. The results of these tests were largely within normal parameters, excluding the common systemic causes of edema. However, the urinalysis yielded atypical findings including pyuria, hematuria, ketonuria, and hemoglobinuria, indicating a possible acute urinary pathology. Additionally, the patient’s glycemic control was brought into question by an elevated HbA1c level, and liver enzyme disturbances were evidenced by increased total and indirect bilirubin, AST, and GGT levels. Serum lipid profile, C-reactive protein (CRP), B-type natriuretic peptide (BNP), and troponin T results were within normal ranges, ruling out cardiovascular causes. With the exclusion of more common etiologies and the temporal association between the initiation of escitalopram and the development of edema, a provisional diagnosis of drug-associated peripheral edema was considered. Escitalopram was subsequently discontinued and resulted in a rapid and complete resolution of edema within three days, further substantiating the causative relationship.

## Patient perspective

Initially, the patient was concerned about the leg edema she started to develop, not sure the reason behind it. Following the identification and cessation of the causative medicine and the initiation of an alternate medication, the patient was happy that her leg edema was resolved quickly and still willing to adhere to her new medication. She was aware of the significance of reporting any worries and not ignoring any signs.

## 4 Discussion

Drug-associated edema refers to the abnormal accumulation of fluid in the interstitial spaces of the body as a result of medication. Although the edema can be frequent with some drugs, it remains inadequately understood and underdiagnosed. This poor characterization concerns both their mechanism and action. And the reporting system for peripheral edema varies from study to study. Medications from different classes have been implicated in causing edema, commonly with anticancer, antihypertensives, corticosteroids, psychotropics, and many more (4). In psychiatry, considering psychotropic drugs and their association with peripheral edema, antipsychotics and antidepressants are mostly reported. The medications with the highest rate of association were mirtazapine, olanzapine, quetiapine, risperidone and pregabalin (5). Four main mechanisms account for the etiology of drug-associated edema: sodium and water retention (renal edema), increased capillary permeability (permeability edema), lymphatic insufficiency (lymphedema), and precapillary arteriolar vasodilation (vasodilatory edema) (4).

The estimated incidence of peripheral edema can vary widely depending on the population studied and the medications involved. For example, peripheral edema is a common side effect of calcium channel blockers (CCBs), with an incidence ranging from 2% to 25% depending on the type of CCB, dosage, and duration of therapy. Amlodipine, in particular, is more likely to lead to peripheral edema compared to nondihydropyridine CCBs and newer lipophilic DHP CCBs (6). Gabapentin is another medication that can cause peripheral edema, reported at an incidence rate of 2% to 8%. The occurrence of edema seems to be dose-related and more common in the elderly population. In a pooled analysis from clinical trials, the incidence increased from 1.4% to 7.5% with doses of 1800 mg/day and up to 12.3% at 3600 mg/day. However, there are cases of edema developing at doses lower than 1800 mg/day, indicating that it might not always be dose-related (7). It’s also important to note that while CCB-associated edema is a frequent issue leading to the use of diuretics, this type of edema is not caused by fluid overload, and using diuretics can pose risks, especially in older adults (6). As for the general prevalence of peripheral edema, one source suggested that approximately 20% of adults older than 50 years may experience edema (8).

In antidepressants, nearly all major classes were found to be associated with edema in a systematic review comparing them. Of these medications, trazodone is the most implicated, followed by mirtazapine in second place and escitalopram in third. Particularly, SSRIs contributed 24.4% compared to the other classes (9). No clear conclusion is made regarding the possible etilology of anti-depressant associated edema, as most studies are case reports, but the proposed etiology involves the antagonism of α1 adrenergic receptors and 5HT2A receptors, leading to vascular smooth muscle relaxation, increased capillary vasodilatation, hydrostatic pressure, and subsequent edema (5) (9). Another possible mechanism suggests that 5-HT1B receptors are in endothelial cells, and stimulation of this receptor by increased serotonin induces vasodilation through the production of nitric oxide (NO) (10). Bilateral leg edema was reported in some cases with the use of escitalopram. Most of the patients were diagnosed with major depressive disorder and started on escitalopram in different doses, with a minimum dose ranging from 10 mg to 30 mg/day as the highest dose reported (11). In our case, the patient was started on 5 mg/day of escitalopram. The duration of the time from starting the medication to reporting the edema ranged between one week to three weeks (12). In our case, similarly, edema was reported 6 days later.

Reviewing the medication history is crucial when the cause of bilateral lower limb edema is unknown. If any medications are suspected to be associated with the edema, they must be stopped, or their dosage reduced. In addition, the basic laboratory work up should focus initially on excluding major systemic diseases, which include heart failure, renal disease, liver disease and DVT. Other possible differential diagnosis include hypothyroidism, lymphedema due to lymphatic obstruction after trauma or surgery, angioedema and urticaria secondary to allergic reaction. Systemic evaluation includes complete blood count, urinalysis, electrolytes, creatinine, blood sugar, thyroid stimulation hormone, albumin, and other tests for specific indication. **Table 1** shows the suggested additional workup for the common differential diagnosis (13).

**Table 1:** Differential diagnosis and suggested work-up for medication-associated lower limb edema.

Differential Diagnosis	Diagnostic studies
Heart failure	ECG, Echocardiogram, chest radiograph, brain natriuretic peptide
Liver disease	ALT, AST, total bilirubin, alkaline phosphatase, prothrombin time, serum albumin
Kidney disease	Urinalysis with exam of sediment, serum lipids
DVT	D-dimer, doppler exam
Lymphedema	Abdominal/pelvic CT scan

In our case, blood tests that include kidney function, liver function, urine analysis, complete blood count, thyroid function test, cardiac tests, and electrolytes showed no significant results or indication of underlying disease. Risk assessment for thrombosis was done which resulted in a very low risk for thrombotic disease. However, thrombotic assessment such duplex ultrasound and D-dimer to rule out deep vein thrombosis (DVT) were not done. Drugs interaction or adverse drug reaction are ruled out because the patient only takes lorazepam before bed (14). Medication associated edema was suspected, and escitalopram was discontinued, the edema resolved after 3 days. Despite escitalopram being a commonly prescribed medication for mood disorders, this case report highlights a rare side effect of the drug, edema. Which emphasizes the need for additional research on the side effects of SSRIs and draws attention to the significance of attentive patient monitoring, educating patients about the potential for edema development even at low therapeutic doses, and promptly reporting any such occurrences.

## 5 Conclusion

Escitalopram associated bilateral leg edema is a side effect that should be considered when prescribing despite its rarity. Edema can occur at low therapeutic doses and in the absence of other possible medical etiologies. This indicates the further need for healthcare professionals to maintain a broad differential diagnosis when encountering peripheral edema, considering drug associated causes in the context of recent medication changes. This shows the importance of close therapeutic monitoring, blood tests, and knowledge of underlying medical issues, drug interactions, and potential adverse effects.

## Data availability statement

The original contributions presented in the study are included in the article/supplementary material. Further inquiries can be directed to the corresponding author.

## Ethics statement

Written informed consent was obtained from the individual(s) for the publication of any potentially identifiable images or data included in this article.

## Author contributions

MHA: Writing – review & editing, Supervision. MA: Writing – review & editing, Writing – original draft, Project administration. SA: Writing – original draft, Writing – review & editing. OS: Writing – original draft, Writing – review & editing. HM: Writing – review & editing.
